# Association of Alzheimer’s disease progression with YKL40 levels in peripheral blood and cerebrospinal fluid: a systematic review and meta-analysis

**DOI:** 10.3389/fneur.2026.1768353

**Published:** 2026-02-05

**Authors:** Qianwen Yang, Ruiqi Wang, Jian Pei, Qinhui Fu, Yijun Zhan, Wenyan Zhu

**Affiliations:** 1 Department of Acupuncture, Longhua Hospital, Shanghai University of Traditional Chinese Medicine, Shanghai, China; 2 International Education College, Shanghai University of Traditional Chinese Medicine, Shanghai, China

**Keywords:** Alzheimer’s disease, mild cognitive impairment, neuroinflammation, pre-AD, YKL40

## Abstract

**Introduction:**

Chitinase 3-like protein 1 (CHI3L1 or YKL40) is a potential neuroinflammatory biomarker linked to the pathogenesis of Alzheimer’s disease (AD). Previous studies have produced inconsistent results regarding YKL40 levels in various clinical stages of AD. This study aims to establish the correlation between YKL40 levels and AD progression through a meta-analysis of YKL40 levels in cerebrospinal fluid (CSF) and peripheral blood.

**Methods:**

Comprehensive searches were conducted in PubMed, Medline, Web of Science, and the Cochrane Library to identify observational studies reporting CSF and peripheral blood YKL40 levels in AD patients, mild cognitive impairment (MCI) patients, preclinical AD (pre-AD) and healthy controls (HCs). A random effects meta-analysis was used to calculate the standardized mean difference (SMD) and 95% confidence intervals (CIs).

**Results:**

Thirty observational studies involving 2,102 AD patients, 1,504 MCI patients, 118 pre-AD individuals, and 2,091 HCs were included. Significant differences in CSF YKL-40 levels were observed in AD vs. HC (SMD = 1.37, 95%CI: [1.09, 1.65]; *p* = 0.000), MCI vs. HC (SMD = 0.96, 95%CI: [0.51, 1.41]; *p* = 0.000), and pre-AD vs. HC (SMD = 0.81, 95%CI: [0.39, 1.22]; *p* = 0.001) comparisons. Peripheral blood YKL-40 levels also demonstrated statistically significant elevations in both AD vs. HC (SMD = 0.40, 95%CI: [0.18, 0.63]; *p* = 0.000) and MCI vs. HC (SMD = 0.79, 95%CI: [0.03, 1.55]; *p* = 0.043) comparisons. However, CSF YKL-40 levels showed no statistically significant difference between AD and MCI groups (SMD = 0.25, 95%CI: [−0.08, 0.57]; *p* = 0.134).

**Discussion:**

Elevated YKL-40 levels in both CSF and peripheral blood are associated with the presence of Alzheimer’s disease and its early stages, indicating that YKL-40 reflects neuroinflammatory processes involved in AD onset. While YKL-40 shows potential value for early identification along the AD continuum, its limited ability to differentiate between MCI and AD highlights the need for its combined use with other biomarkers in disease staging and progression assessment.

**Systematic review registration:**

https://www.crd.york.ac.uk/PROSPERO/view/CRD420251031837.

## 1 Introduction

With the evolving AT (N) diagnostic framework, “inflammatory/immune mechanisms (I)” has been formally integrated into the biomarker system for Alzheimer’s disease (AD) as biomarkers of non-specific processes involved in AD pathophysiology (1). In recent years, neuroinflammation has been identified as a central player in the pathological cascade of AD. From a mechanistic taxonomy perspective, this process of neuroinflammatory action comprises two distinct cellular response modules: astrocyte activation and microglial reactivity (2). As a protein predominantly derived from astrocytes, chitinase 3-like protein 1 (CHI3L1), also known as YKL-40, has been widely regarded as a biomarker associated with neuroinflammatory processes, which are thought to play an important role in the pathogenesis of cognitive decline in AD (3). However, the temporal dynamics of this marker in longitudinal disease monitoring remain contentious, and its discriminative efficacy across AD clinical stages requires further studies.

In the pathological cascade of AD, cerebrospinal fluid (CSF) YKL-40 has been proposed as a key indicator of neuroinflammatory activity, with its dynamic expression closely associated with disease stages and pathological patterns. Evidence indicates that YKL-40 levels increase with advancing age (4), and are elevated in patients with AD (5, 6). In addition, a significant distinction in CSF YKL-40 levels was observed when comparing mild cognitive impairment (MCI) patients to the AD cohort, suggesting that CSF YKL-40 may reflect disease progression (7, 8). Regarding the temporal utility of biomarkers, longitudinal cohort studies show that YKL-40 release into CSF occurs at a later stage within the AD pathological cascade, indicating that CSF YKL-40 is not a presymptomatic biomarker for early-stage AD, which is more applicable for monitoring disease progression rather than preclinical screening (9). Recent studies focusing on YKL-40 in the progression of AD have advanced rapidly. A large community-based cohort study including over 6,000 participants reported associations between elevated plasma YKL-40 levels and lower brain volume, worse cognitive performance, and a higher risk of dementia. In addition, plasma YKL-40 levels were shown to correlate with cerebrospinal fluid YKL-40 concentrations, and both were associated with progression from MCI to dementia (10). However, existing meta-analyses have reported inconsistent findings regarding peripheral blood YKL-40 levels in AD. A meta-analysis found no statistically significant difference in peripheral blood YKL-40 levels between AD and non-dementia groups (11).

Previous systematic reviews on YKL-40 have largely considered it as one component within broader biomarker panels (8, 12–15), or have restricted their analyses to comparisons between AD patients and healthy control groups (11). However, changes in YKL-40 levels across the entire AD continuum—particularly during the preclinical stage—have not been systematically quantified. In addition, direct parallel comparisons of YKL-40 levels between CSF and peripheral blood remain scarce.

Therefore, the present study aimed to systematically quantify alterations in YKL-40 levels across the AD continuum, including preclinical AD, mild cognitive impairment, and AD dementia, through a parallel meta-analysis of cerebrospinal fluid and peripheral blood measurements. It combines up-to-date data from relevant observational studies to test the hypothesis that elevated YKL-40 levels are associated with cognitive decline, thereby systematically assessing its potential as a biological indicator for monitoring disease advancement.

## 2 Methods

The protocol is registered at the International Prospective Register of Systematic Reviews (PROSPERO) (registration number: CRD420251031837).

### 2.1 Search strategy

Two investigators (WR and ZW) conducted independent searches in the PubMed, Medline, Web of Science, and the Cochrane Library databases for articles published up to 1st May 2025. The main keywords included (YKL40 OR CHI3L1) AND (Alzheimer’s Disease OR Senile Dementia OR AD OR Mild Cognitive Impairment OR MCI OR preclinical AD OR pre-AD) (Supplementary Table S1).

### 2.2 Selection criteria

The inclusion criteria were: (1) studies assessing CSF or blood (plasma or serum) YKL40 in AD patients, MCI patients, pre-AD, and HCs. (2) Utilization of international diagnostic criteria for AD, MCI, and pre-AD was reported. (3) Availability of YKL40 data for both disease and control groups. The exclusion criteria included (1) studies lacking precise YKL40 levels, even after contact with the corresponding author; and (2) reviews, abstracts, case reports, letters, and commentaries.

### 2.3 Data extraction

A dual independent review process was implemented following a predefined protocol, wherein two trained investigators systematically identified and screened eligible studies. Data extraction was performed using standardized templates capturing nine critical parameters: the first author’s name, publication year, country of origin, sample size, diagnostic criteria implementation, mean age, gender composition, YKL-40 biomarker concentrations in either CSF or peripheral blood, and assay method. For studies reporting range-based data, parametric approximation methods were applied to derive means and standard deviations (SD) in accordance with Cochrane Handbook guidelines. All extracted variables were systematically archived in standardized spreadsheets to ensure data integrity.

### 2.4 Quality assessment

The methodological quality of observational studies was rigorously assessed using the Newcastle-Ottawa Scale (NOS), a validated tool comprising three methodological domains across eight specific items, with a maximum attainable score of 9. Studies achieving ≥7 points were classified as high-quality based on established thresholds (16). Dual independent evaluation was conducted by two trained assessors (WR and ZW) using standardized NOS protocols, with discrepancies resolved through consensus-building consultation with a third senior researcher (PJ).

### 2.5 Statistical analysis

All statistical analyses were performed using STATA version 18.0 (StataCorp LLC, College Station, TX, United States). The pooled standardized mean difference (SMD) with corresponding 95% confidence intervals (CI) was calculated to compare YKL-40 levels between AD-related cognitive impairment patients and cognitively intact healthy controls. Interstudy heterogeneity was quantified through Higgins *I^2^* statistics derived from Cochrane’s Q-test. A random-effects model was employed when *I^2^* ≥ 50%, indicating substantial heterogeneity, whereas a fixed-effects model was adopted for lower heterogeneity levels. To investigate potential sources of heterogeneity, predefined subgroup analyses and meta-regression were conducted focusing on three covariates: mean age, diagnostic criteria implementation, and female participant proportion. Sensitivity analysis was performed through iterative exclusion of individual studies to verify the robustness. Publication bias was assessed using Egger’s regression test complemented by funnel plot asymmetry evaluation. Statistical significance threshold was set at *p* < 0.05 (two-tailed).

## 3 Results

### 3.1 Literature selection

**Figure 1:**
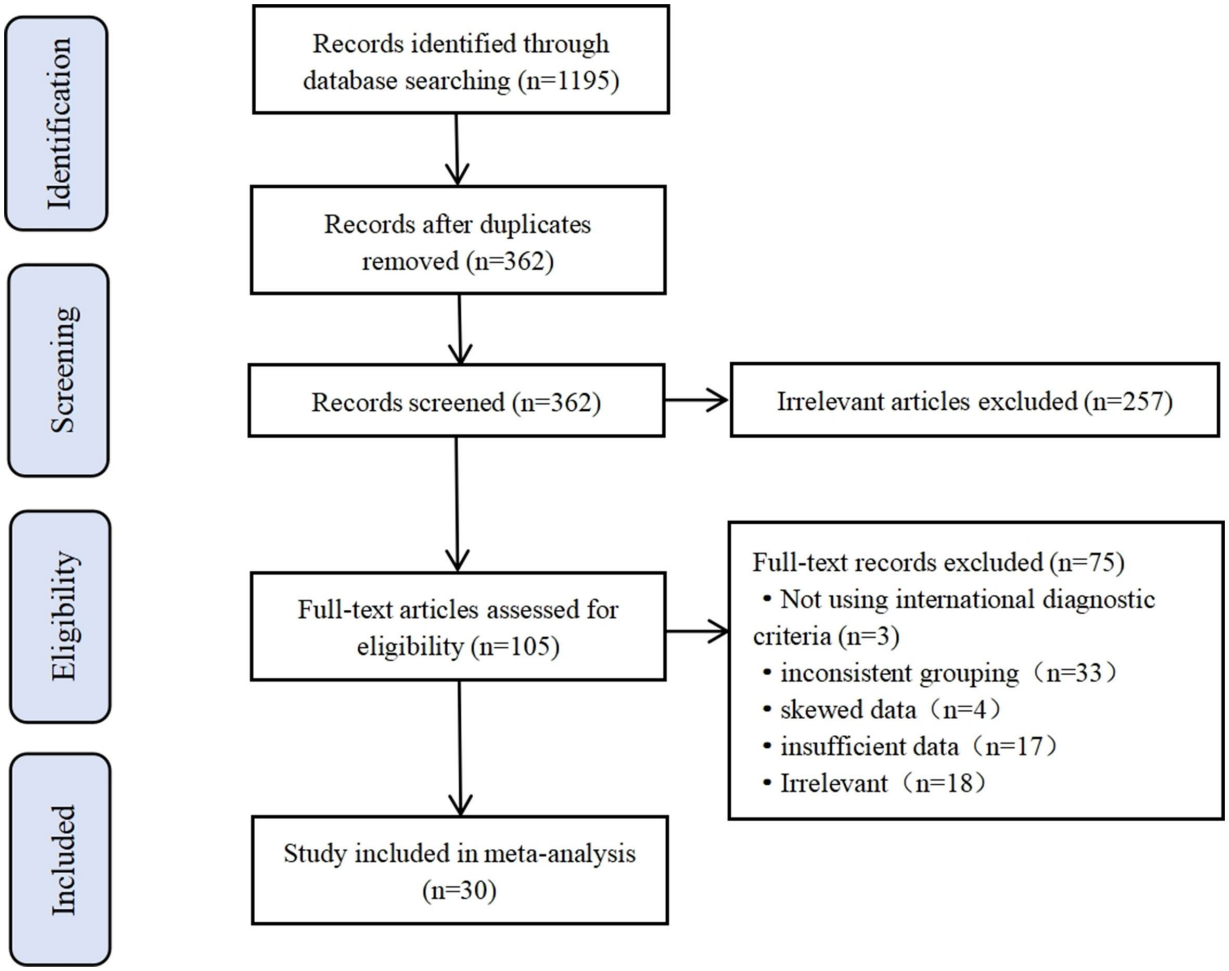
Flow chart of the study selection process and results.

Following a systematic database search using predefined search terms, 1,195 potentially relevant articles were initially identified, including 256 in PubMed, 242 in Medline, 43 in Cochrane, and 444 in Web of Science. After rigorous removal of duplicate records through automated and manual verification processes, 362 unique articles remained eligible for evaluation. Subsequent preliminary screening based on titles and abstracts excluded 257 publications that failed to meet thematic relevance criteria, resulting in 105 articles progressing to full-text assessment. After reviewing full-text context of the remaining 105 articles, 75 articles were excluded as follows: 3 articles were removed due to absence of international diagnostic criteria, 33 studies were excluded for inconsistent grouping, 4 articles demonstrated skewed data distribution, 17 studies were eliminated for incomplete datasets, and 18 publications were deemed irrelevant to the research theme. After rigorous screening, 30 articles met all inclusion criteria and were subsequently incorporated into the systematic analysis, which is presented in Figure 1.

### 3.2 Study characteristics

The thirty articles (5, 17–45) were all published between 2011 and 2025, with a concentration of publications occurring from 2019 to 2023. The 30 included studies involving 5,815 participants stratified into distinct diagnostic categories: 2,102 AD patients, 1,504 MCI cases, 118 pre-AD individuals, and 2,091 HCs. Biomarker quantification revealed a differential analytical focus across studies, with 8 investigations reporting YKL-40 levels in peripheral blood specimens and 24 studies providing CSF measurements. Regarding methodological approaches, enzyme-linked immunosorbent assay (ELISA) was employed as the primary detection method in 28 studies, while the remaining two studies utilized the Elecsys electrochemiluminescence immunoassay and Simple-Plex microfluidic immunoassay system, respectively. Quality assessment using the NOS revealed scores ranging from 6 to 8, with 28 studies meeting high-quality criteria and 2 studies classified as moderate quality (Supplementary Tables S2, S3).

### 3.3 Association between CSF levels of YKL-40 and AD progression

#### 3.3.1 Meta-analysis of CSF levels of YKL-40 between AD and HCs

**Figure 2:**
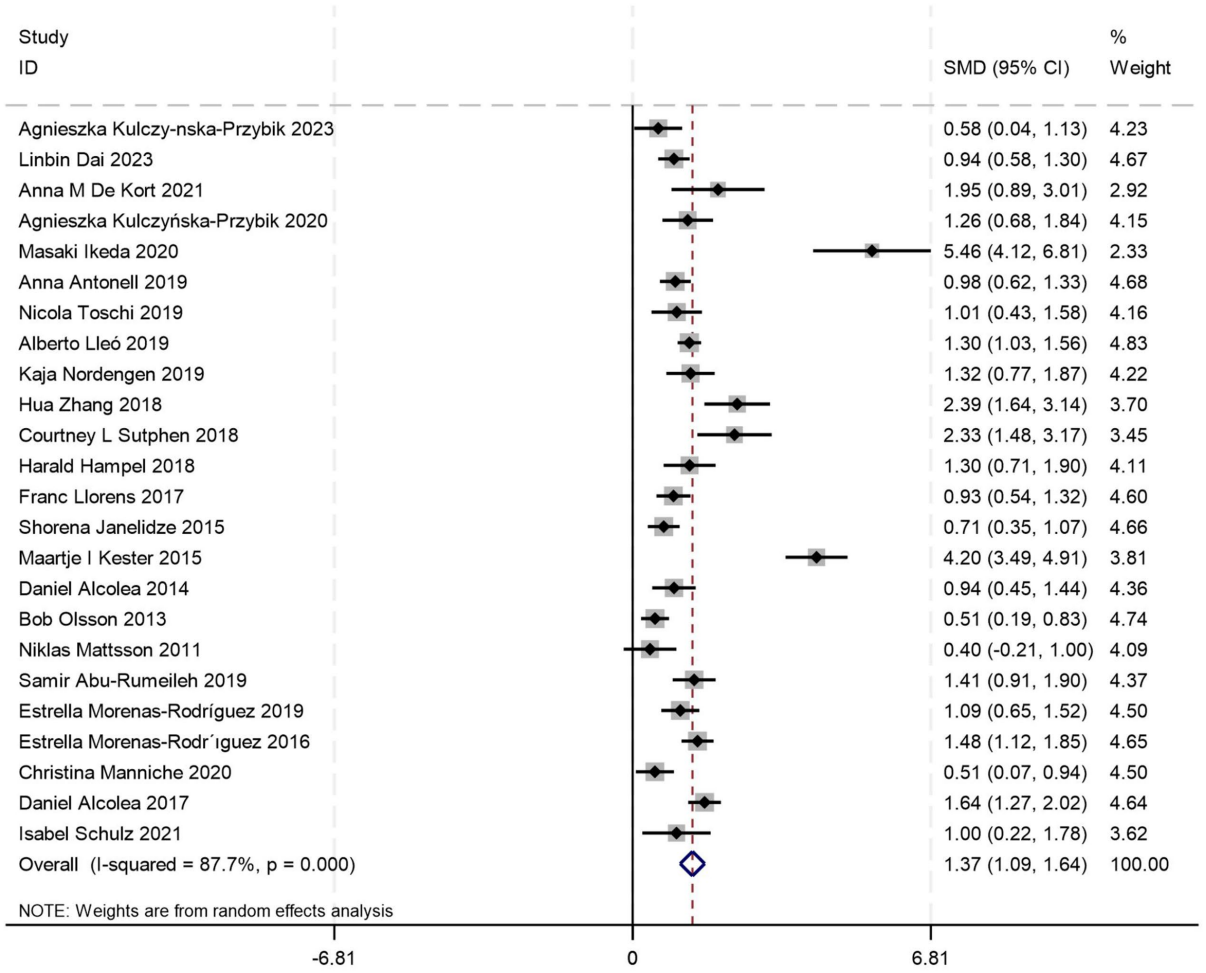
Forest plot of CSF YKL40 levels in patients with AD compared to controls.

Twenty-four (5, 18, 21, 23–29, 31–43, 45) studies provided analyzable data comparing CSF YKL-40 levels between AD patients and HCs. Pooled analysis demonstrated significantly elevated CSF YKL-40 levels in AD patients versus HC [SMD = 1.37, 95%CI: (1.09, 1.65); *p* = 0.000; *I^2^* = 87.7%, *p* = 0.000] (Figure 2).

Because of substantial heterogeneity, we conducted stratified subgroup analyses based on three aspects to investigate the underlying drivers of this heterogeneity systematically: (1) diagnostic criteria implementation for AD, (2) mean age of AD cohorts, and (3) female proportion within AD patient populations. These methodological approaches aimed to identify the influential moderators contributing to heterogeneity. Among these, two studies did not report age data, and one study did not provide information on gender distribution. Subgroup analyses stratified by the aforementioned three factors consistently demonstrated that CSF YKL-40 levels in AD patients were significantly higher than those in HCs across all subgroups (all *p* = 0.000) (Table 1). Nevertheless, substantial residual heterogeneity persisted across all subgroups (*I^2^* range 80.5–92.9%). Meta-regression modeling indicated that these factors do not fully explain the sources of heterogeneity, suggesting undisclosed moderators influence biomarker variability.

**Table 1:** Subgroup analysis of CSF YKL-40 levels with AD.

Subgroups	n of studies	SMD (95%CI)	*I* ^2^	*P*-value
Age (years)				
≧70	13	1.13 (0.83, 1.44)	80.50%	0.000
<70	9	1.88 (1.29, 2.47)	92.90%	0.000
Diagnostic criteria				
NIA-AA	12	1.27 (0.95, 1.58)	83.20%	0.000
NINCDS-ADRDA	11	1.47 (0.91, 2.02)	91.60%	0.000
IWG-2	1	1.41 (0.92, 1.90)	NA	0.000
Proporption of female (%)				
≧50	16	1.31 (0.99, 1.63)	85.40%	0.000
<50	7	1.49 (0.86, 2.13)	92.60%	0.000

#### 3.3.2 Meta-analysis of CSF levels of YKL-40 between MCI and HCs

**Figure 3:**
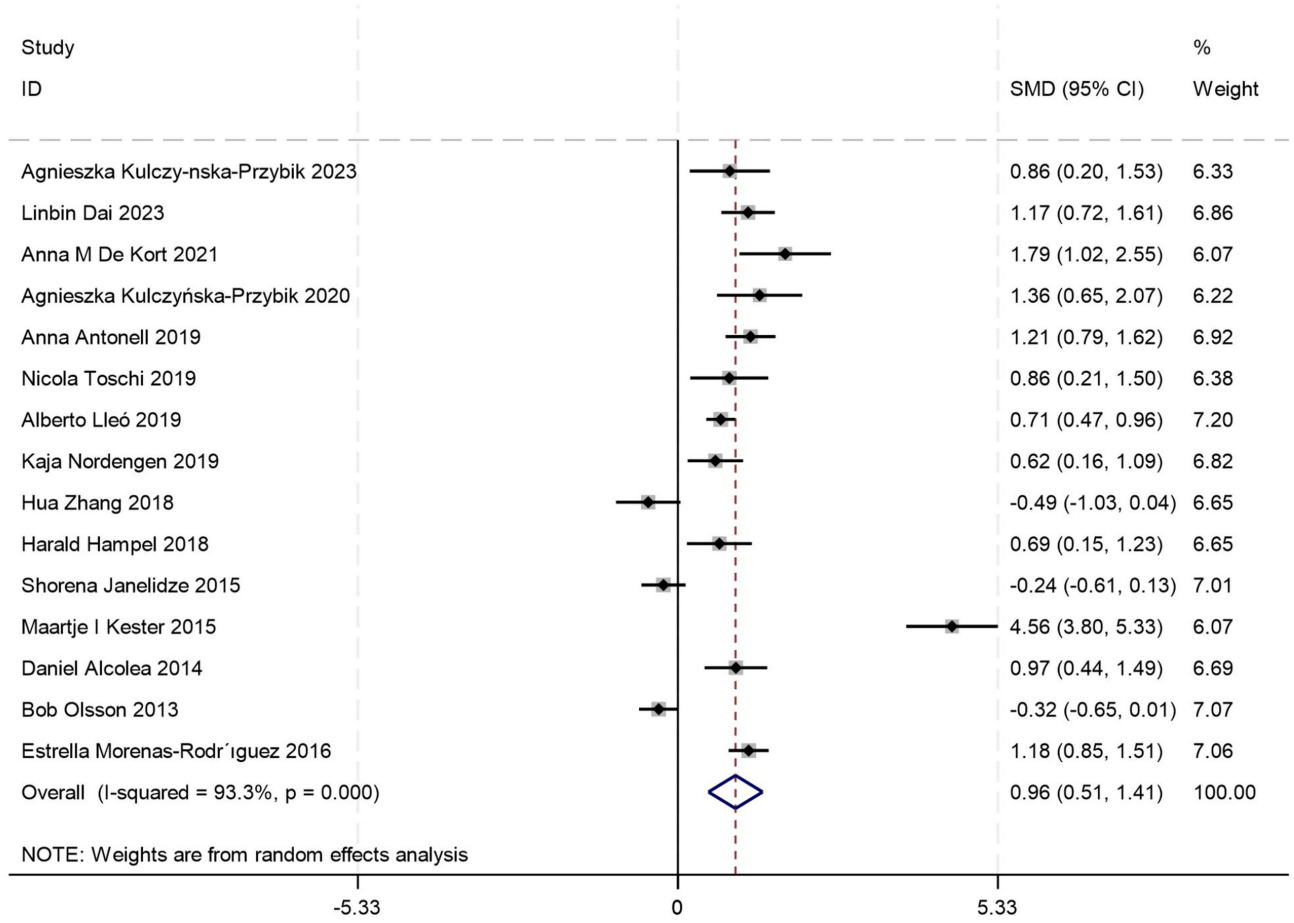
Forest plot of CSF YKL40 levels in patients with MCI compared to controls.

There are 15 eligible studies (5, 18, 21, 24, 26, 28, 29, 31, 34, 35, 38–41, 43) investigating CSF YKL-40 levels between MCI patients and HCs. Quantitative synthesis revealed a statistically significant elevation in CSF YKL-40 levels among MCI patients compared to HCs [SMD = 0.96, 95%CI: (0.51, 1.41); *p* = 0.000; *I^2^* = 93.3%, *p* = 0.000] (Figure 3).

Given substantial between-study heterogeneity (*I^2^* = 92.4%), subgroup analyses were stratified by diagnostic criteria (NIA-AA vs. NINCDS-ADRDA), age cutoff (≥70 vs. <70 years), and gender composition (proportion of female participants ≥50% vs. <50%). Among these, two studies did not report age data, and one study did not provide information on gender distribution. The stratified analysis revealed no significant difference in CSF YKL-40 levels between MCI patients and HCs within the ≥70 years subgroup [SMD = 0.55, 95%CI: (−0.12, 1.21); *p* = 0.105; *I^2^* = 89.5%, *p* = 0.000] (Table 2). However, statistically significant elevations were observed in MCI patients across the remaining subgroups (Table 2). Notably, while the NIA-AA subgroup demonstrated moderate heterogeneity (*I^2^* = 46.0%), all other subgroups maintained high heterogeneity indices (>75%). This pattern suggests diagnostic criteria may partially account for the observed heterogeneity, though residual confounding factors warrant further investigation through meta-regression analysis.

**Table 2:** Subgroup analysis of CSF YKL-40 levels with MCI.

Subgroups	n of studies	SMD (95%CI)	*I* ^2^	*P*-value
Age (years)				
≧70	6	0.55 (−0.12, 1.21)	89.5%	0.105
<70	7	1.26 (0.57, 1.96)	95.6%	0.000
Diagnostic criteria				
NIA-AA	7	0.99 (0.77, 1.20)	46.0%	0.000
NINCDS-ADRDA	8	0.94 (0.04, 1.84)	96.0%	0.040
Proporption of female (%)				
≧50	7	0.70 (0.11, 1.29)	92.4%	0.020
<50	7	1.18 (0.38, 1.98)	94.9%	0.004

#### 3.3.3 Meta-analysis of CSF levels of YKL-40 between pre-AD and HCs

**Figure 4:**
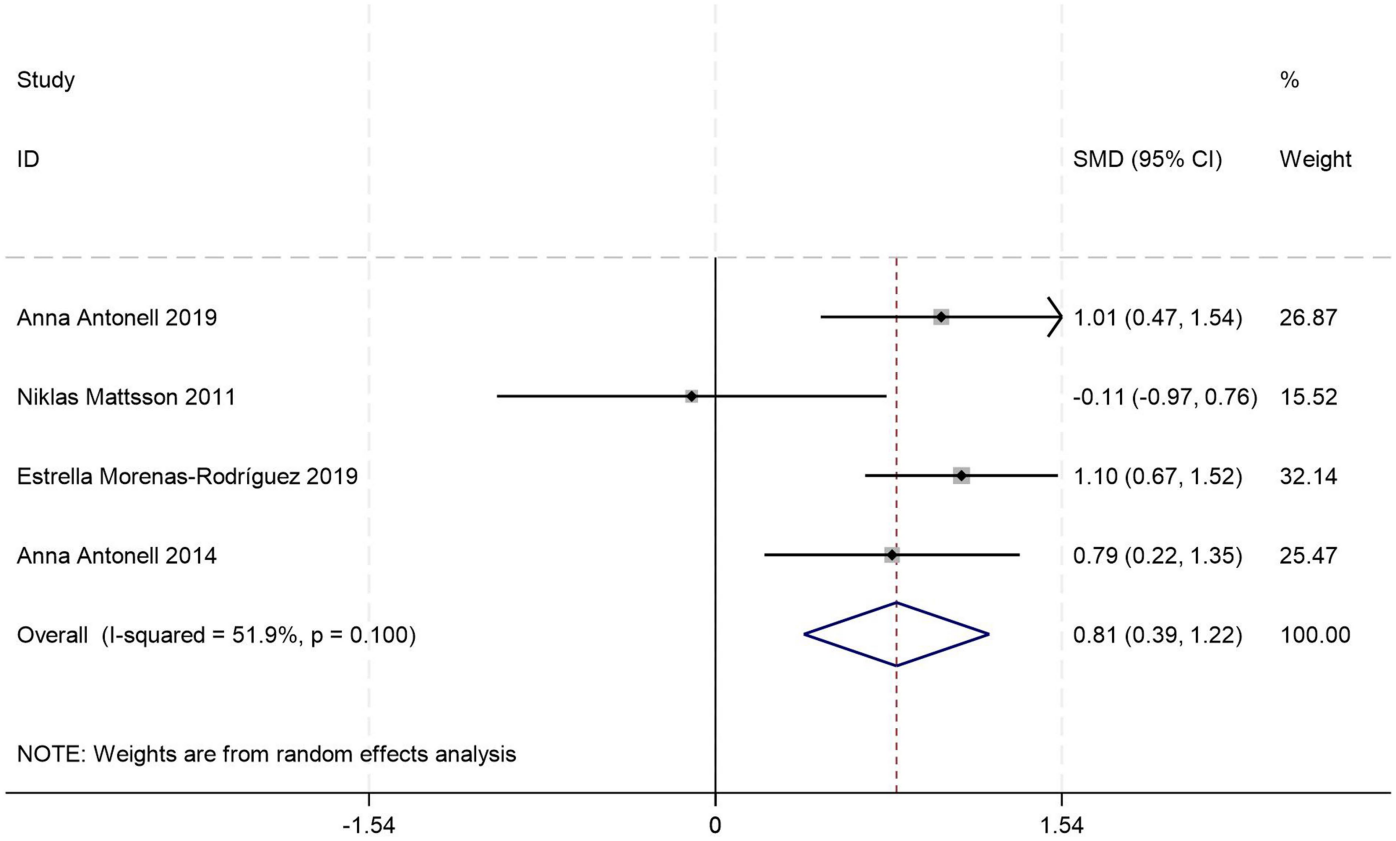
Forest plot of CSF YKL40 levels in patients with pre-AD compared to controls.

This meta-analysis pooled data from four case–control studies (26, 33, 42, 45) comparing CSF YKL-40 levels between pre-AD subjects and HCs. Quantitative synthesis demonstrated significantly elevated CSF YKL-40 concentrations in the pre-AD group compared to HCs with moderate heterogeneity across studies [SMD = 0.81, 95%CI: (0.39, 1.22); *p* = 0.001; *I^2^* = 51.9%, *p* = 0.100] (Figure 4).

To investigate sources of heterogeneity, subgroup analyses were conducted based on diagnostic criteria, age stratification, and female proportion. Among the results of diagnostic criteria and gender stratification, the distribution of studies was consistent. In the subgroup that followed the NINCDS—ADRDA criteria and had a female proportion less than 50%, there was only the same paper [by Niklas Mattsson et al. (45)], and in the pre-AD group, the CSF YKL40 level had no significant difference from that of the HC group. The remaining subgroups all showed that the CSF YKL40 level in the pre-AD group was significantly higher than that in the HC group. It can be seen that this essay is the main source of heterogeneity in this meta-analysis. However, due to the relatively small amount of data included in general, it is impossible to directly determine the impact of age, diagnostic criteria, and gender distribution on the results (Table 3).

**Table 3:** Subgroup analysis of CSF YKL-40 levels with pre-AD.

Subgroups	n of studies	SMD (95%CI)	*I* ^2^	*P*-value
Age (years)				
≧70	3	0.69 (0.10, 1.29)	66.4%	0.023
<70	1	1.01 (0.47, 1.54)	NA	0.000
Diagnostic criteria				
NIA-AA	3	0.99 (0.70, 1.28)	0.0%	0.000
NINCDS-ADRDA	1	−0.11 (−0.97, 0.76)	NA	0.812
Proporption of female (%)				
≧50	3	0.99 (0.70, 1.28)	0.0%	0.000
<50	1	−0.11 (−0.97, 0.76)	NA	0.812

#### 3.3.4 Meta-analysis of CSF levels of YKL-40 between AD and MCI

**Figure 5:**
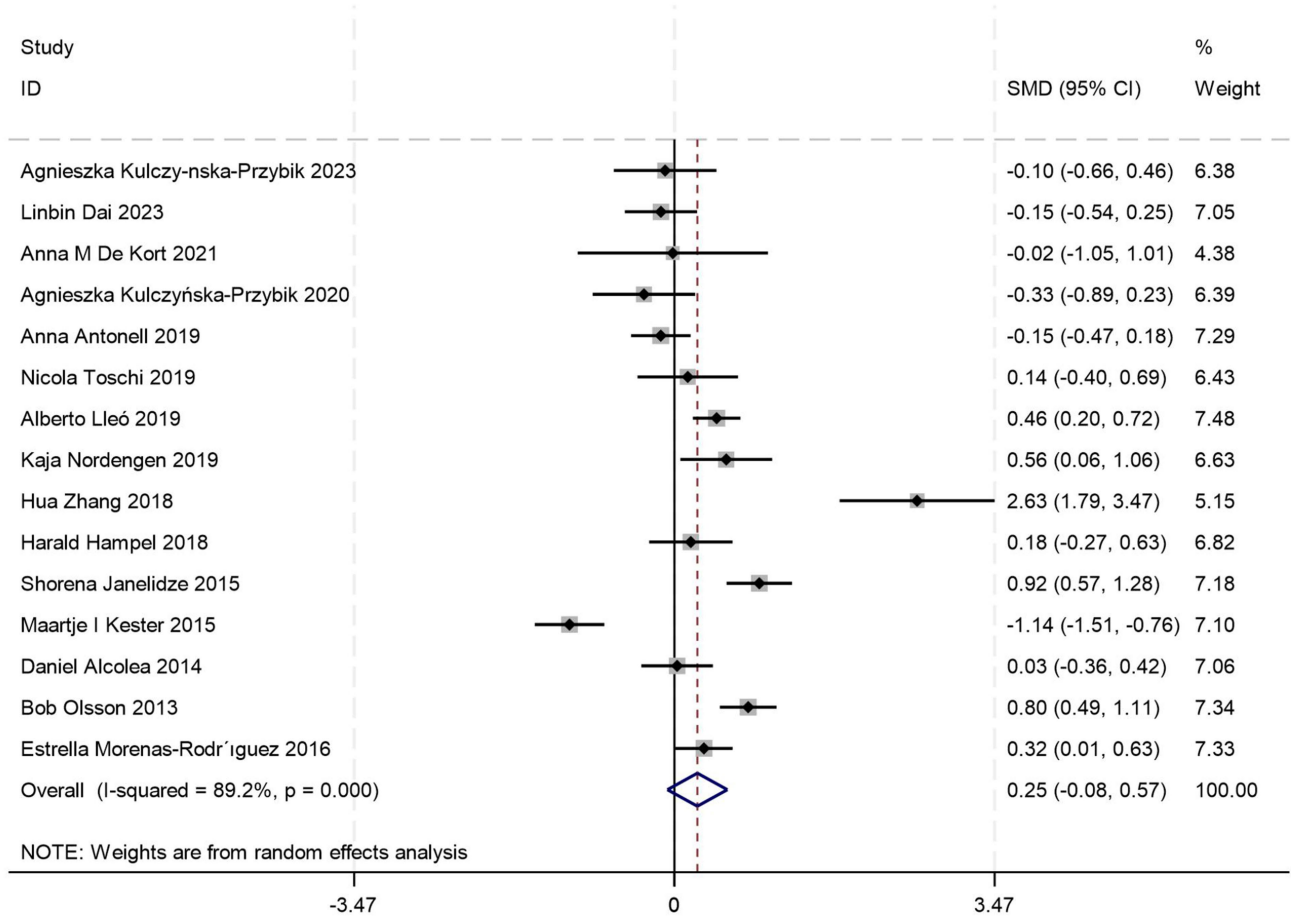
Forest plot of CSF YKL40 levels in patients with AD compared to MCI.

Fifteen comparative studies (5, 18, 21, 24, 26, 28, 29, 31, 34, 35, 38–41, 43) comparing CSF YKL-40 levels between AD patients and those with MCI were included. Quantitative analysis demonstrated comparable CSF YKL-40 concentrations between AD and MCI cohorts (*p* = 0.12, Student’s *t*-test), suggesting no pathophysiological distinction in this biomarker profile [SMD = 0.25, 95%CI: (−0.08, 0.57); *p* = 0.134; *I^2^* = 89.2%, *p* = 0.0004] (Figure 5).

To address substantial between-study heterogeneity, stratified analyses were conducted based on diagnostic criteria, age stratification, and female proportion. Among these, two studies did not report age data, and one study did not provide information on gender distribution. Age-stratified analyses revealed: no significant difference in CSF YKL-40 levels between AD and MCI patients in the ≥70 years subgroup [SMD = 0.55, 95%CI: (−0.12, 1.21); *p* = 0.105; *I^2^* = 89.5%, *p* = 0.000] (Table 4), whereas the <70 years subgroup showed marked elevation in AD (*p* = 0.000) (Table 4), suggesting age stratification may account for a source of high heterogeneity. Stratified analyses based on diagnostic criteria and female proportion revealed significantly elevated CSF YKL-40 levels in AD patients compared to those with MCI (all *p* < 0.05) (Table 4), whereas the overall meta-analysis incorporating all studies failed to reach significance (*p* = 0.134) (Figure 5), suggesting that different diagnostic criteria and imbalanced gender distribution may serve as confounding factors contributing to the high heterogeneity observed in this study.

**Table 4:** Subgroup analysis of CSF YKL-40 levels with AD compared to MCI.

Subgroups	n of studies	SMD (95%CI)	*I* ^2^	*P*-value
Age (years)				
≧70	8	0.55 (−0.12, 1.21)	89.5%	0.105
<70	5	1.26 (0.57, 1.96)	95.6%	0.000
Diagnostic criteria				
NIA-AA	7	0.99 (0.77, 1.20)	46.0%	0.000
NINCDS-ADRDA	8	0.94 (0.04, 1.84)	96.0%	0.040
Proporption of female (%)				
≧50	10	0.70 (0.11, 1.29)	92.4%	0.020
<50	4	1.18 (0.38, 1.98)	94.9%	0.004

### 3.4 Association between peripheral blood levels of YKL-40 and AD progression

#### 3.4.1 Meta-analysis of peripheral blood levels of YKL-40 between AD and HCs

**Figure 6:**
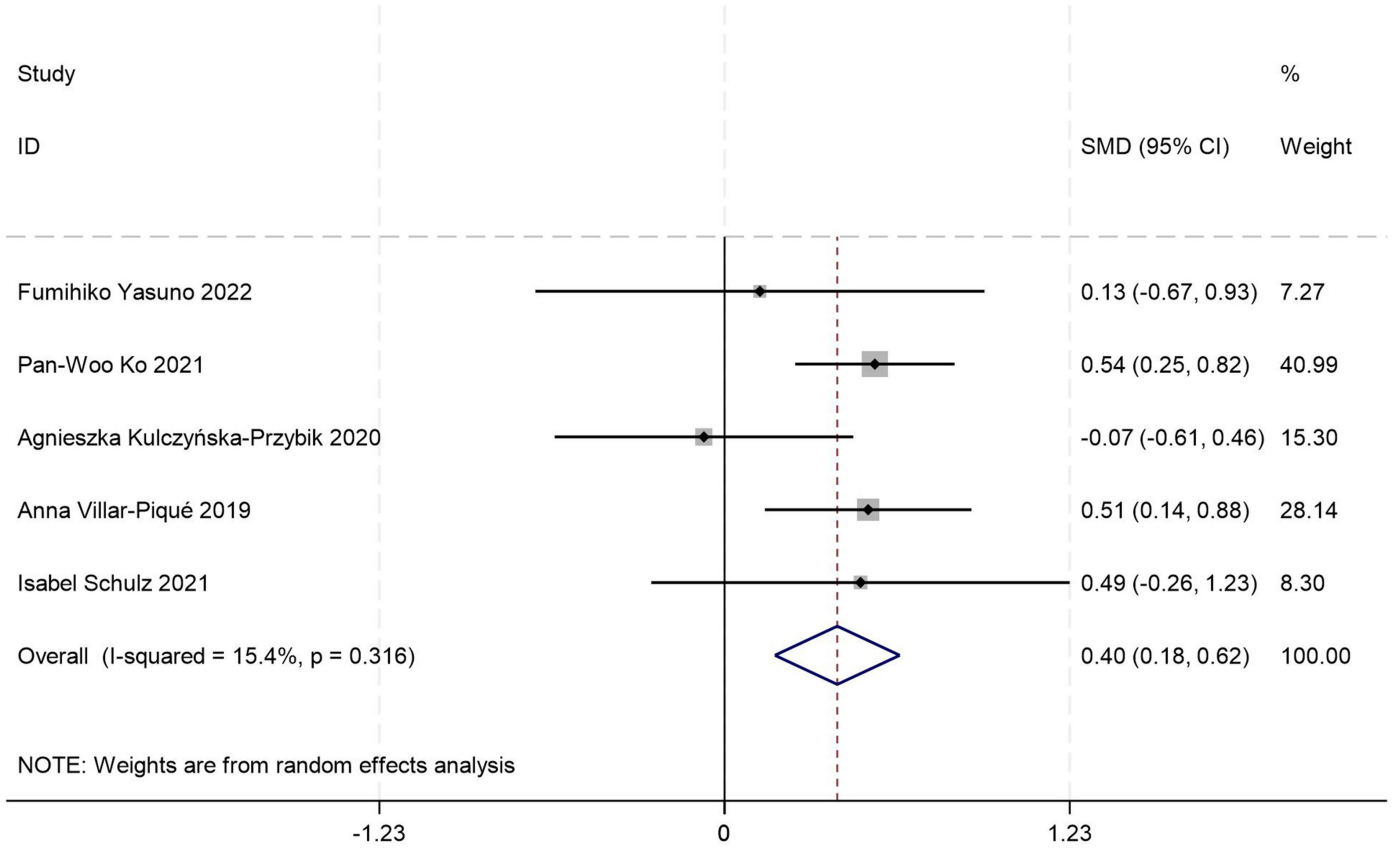
Forest plot of peripheral blood YKL40 levels in patients with AD compared to HC.

This meta-analysis synthesized five case–control studies (20, 22–24, 30) comparing peripheral blood YKL-40 levels between AD patients and HCs. Random-effects modeling revealed significantly elevated YKL-40 concentrations in AD patients versus HCs [SMD = 0.40, 95%CI: (0.18, 0.63); *p* = 0.000; *I^2^* = 15.4%, *p* = 0.316] (Figure 6).

All included studies adhered to the NIA-AA diagnostic criteria, with subgroup analyses stratified by age distribution, gender distribution, and peripheral blood sample type (serum vs. plasma). One study failed to report age, gender data, or specify blood sample type. Stratified analyses revealed complete overlap in study inclusion between the age >70 years subgroup and serum subgroup, demonstrating no significant difference in serum YKL-40 levels between AD patients and HCs (*p* = 0.253) (Table 5). Conversely, identical data inclusion was observed between the age ≤70 years subgroup and plasma subgroup, where AD patients exhibited significantly elevated plasma YKL-40 levels compared to HCs (*p* = 0.000) (Table 5). These findings suggest potential interactive effects between age stratification and blood matrix type on YKL-40 measurements. However, the limited total sample size and complete collinearity between subgroups precluded quantification of independent contributions from these factors to the effect magnitude. Gender-stratified analysis demonstrated statistically significant YKL-40 level differences between AD patients and HCs in female-predominant subgroups (≥50%; *p* = 0.003) (Table 5), whereas no significant differences were observed in female-minority subgroups (<50%; *p* = 0.203) (Table 5).

**Table 5:** Subgroup analysis of peripheral blood YKL-40 levels with AD compared to HC.

Subgroups	n of studies	SMD (95%CI)	*I* ^2^	*P*-value
Age (years)				
≧70	2	0.32 (−0.23, 0.86)	0.0%	0.253
<70	2	0.53 (0.30, 0.75)	0.0%	0.000
Proporption of female (%)				
≧50	3	0.50 (0.28, 0.72)	0.0%	0.003
<50	1	0.49 (−0.26, 1.23)	NA	0.203
Blood				
Plasma	2	0.53 (0.30, 0.75)	0.0%	0.000
Serum	2	0.32 (−0.23, 0.86)	0.0%	0.253

#### 3.4.2 Meta-analysis of peripheral blood levels of YKL-40 between MCI and HCs

**Figure 7:**
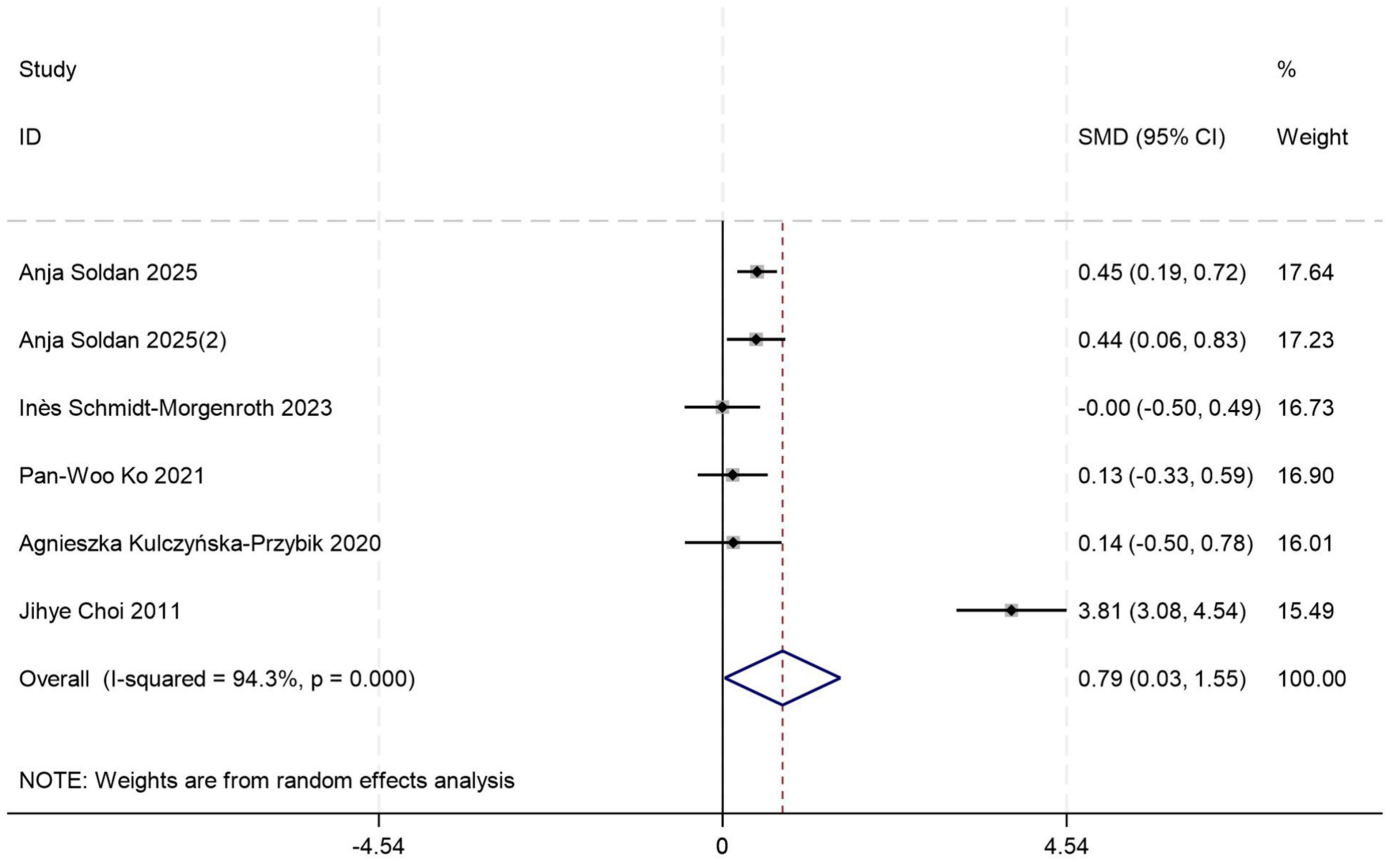
Forest plot of peripheral blood YKL40 levels in patients with MCI compared to controls.

Five sets of control data (17, 19, 22, 24, 44) were included in the analysis comparing peripheral blood YKL-40 levels between MCI patients and HCs. Random-effects modeling demonstrated significantly elevated YKL-40 concentrations in MCI patients versus HCs [SMD = 0.79, 95%CI: (0.03, 1.55); *p* = 0.043; *I^2^* = 94.3%, *p* = 0.000] (Figure 7).

Given substantial between-study heterogeneity, stratified subgroup analyses identified blood sample types and diagnostic criteria as potential contributors to heterogeneity, which may be limited by small sample size. Out of these, the plasma-based subgroup (*p* = 0.035) (Table 6) showed higher effect magnitudes compared to the serum-based subgroup (*p* = 0.984) (Table 6). Both NIA-AA (*p* = 0.000) (Table 6) and NINCDS-ADRDA (*p* = 0.000) (Table 6) subgroups consistently demonstrated significant differences in peripheral blood YKL-40 levels between MCI patients and HCs.

**Table 6:** Subgroup analysis of peripheral blood YKL-40 levels with MCI compared to HCs.

Subgroups	n of studies	SMD (95%CI)	*I* ^2^	*p*-value
Diagnostic criteria				
NIA-AA	5	0.32 (0.15, 0.50)	0.0%	0.000
NINCDS-ADRDA	1	3.81 (3.08, 4.54)	NA	0.000
Blood				
Plasma	4	1.16 (0.08, 2.23)	96.3%	0.035
Serum	1	−0.01 (−0.50, 0.49)	NA	0.984

In short, given the limited number of included studies and varying sample sizes across cohorts, large-scale studies are required to identify the discriminative capacity of peripheral blood YKL-40 levels for AD staging.

### 3.5 Sensitivity analysis and publication bias

**Figure 8:**
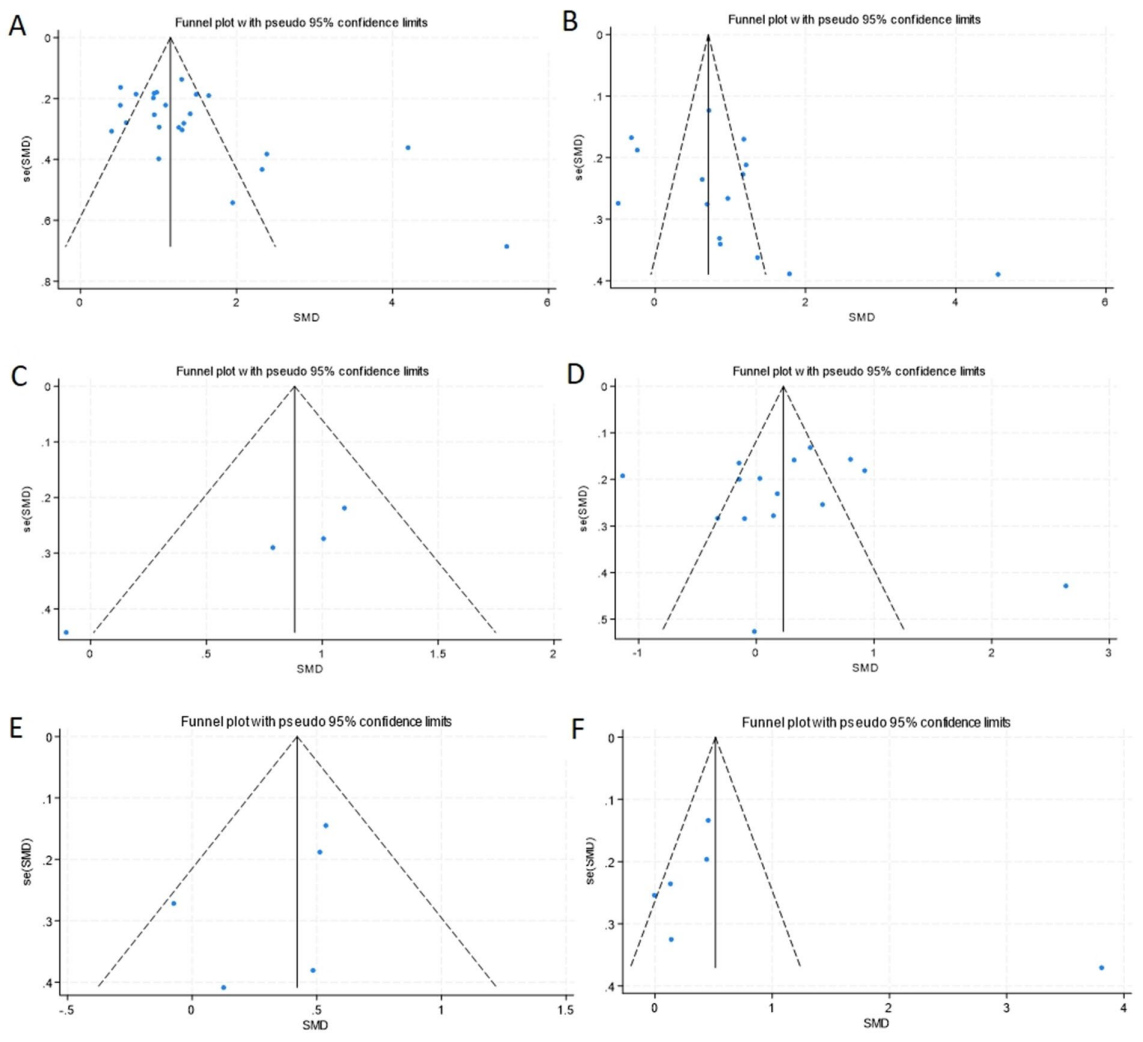
Funnel plot of YKL40 levels in different groups. **(A)** Funnel plot of CSF YKL-40 levels with AD compared to HCs. **(B)** Funnel plot of CSF YKL-40 levels with MCI compared to HCs. **(C)** Funnel plot of CSF YKL-40 levels with pre-AD compared to HCs. **(D)** Funnel plot of CSF YKL-40 levels with AD compared to MCI. **(E)** Funnel plot of peripheral blood YKL40 levels in patients with AD compared to HCs. **(F)** Funnel plot of peripheral blood YKL40 levels in patients with MCI compared to HCs.

Sensitivity analysis employing leave-one-out methodology demonstrated negligible fluctuations in pooled effect estimates, confirming the robustness of primary outcomes. Publication bias was systematically assessed across all groups. Egger’s regression test revealed significant publication bias in CSF YKL-40 comparisons between AD patients and HCs (*p* = 0.017), while no statistically significant bias risk was detected in other subgroups (all *p* > 0.05), which were corroborated through the funnel plot test (Figure 8).

## 4 Discussion

### 4.1 Summary of findings

Previous studies have demonstrated that incorporating CSF inflammatory biomarkers into the AD biomarker panel enhances diagnostic accuracy for clinical staging at baseline and improves the predictive value of AD biomarkers for cognitive decline (46), highlighting the critical role of sensitive inflammatory biomarkers in disease prognostication. YKL-40, a potential biomarker of neuroinflammation and astrocyte activation, exhibits significant disease-promoting effects during the early pathological progression of AD (47). The current study demonstrated statistically significant elevations in CSF YKL-40 levels in AD, MCI, and pre-AD groups compared with HCs (all *p* < 0.05), with peripheral blood YKL-40 levels also showing significant differences in AD and MCI groups (all *p* < 0.05). Notably, while CSF YKL-40 levels in AD patients showed an increasing trend compared to MCI patients, this difference did not reach statistical significance (*p* = 0.53).

These findings are consistent with and extend previous meta-analyses. Earlier studies consistently reported elevated CSF YKL-40 levels in AD, but showed inconsistent or no significant differences in peripheral blood (11). Other meta-analyses, often based on a limited number of studies, reported higher CSF YKL-40 levels in AD than in MCI, with no significant difference between MCI and controls (7). By synthesizing more recent evidence, our updated analysis suggests that YKL-40 elevations may already be present at the MCI and even preclinical stages, thereby observed differences between MCI and AD in earlier reports. Emerging evidence further suggests a stage-dependent discriminative value of YKL-40, particularly in early MCI (48). Despite heterogeneity across studies regarding YKL-40’s efficacy in distinguishing AD stages, our findings support its potential as a predictive biomarker for AD progression.

In subgroup analyses of CSF YKL-40 levels stratified by age, diagnostic criteria, and female proportion, we observed no significant differences in CSF YKL-40 levels between MCI patients aged >70 years and either AD patients or HCs. Comparisons between pre-AD and HCs within the NINCDS-ADRDA diagnostic criteria subgroup and the subgroup with <50% female participants also showed non-significant differences, likely attributable to limited sample sizes. Crucially, age and sex emerge as critical modulators of YKL-40 levels: cumulative evidence demonstrates age-dependent elevation of CSF YKL-40 that correlates with aging progression (49, 50)^.^ A meta-analysis revealed associations between female proportion and YKL-40 levels in AD/MCI cohorts (51). Mechanistic studies suggest sex-specific interactions, with females exhibiting more pronounced p-tau181 elevation than males at equivalent YKL-40 levels (52). Gene expression profiling further indicates heightened YKL-40 transcription in female AD brains compared to males (53). However, conflicting data exist regarding peripheral blood YKL-40, with a study showing plasma YKL-40 levels independent of age and sex (54).

For peripheral blood YKL-40 analyses, stratification by blood sample type was performed in addition to age, diagnostic criteria, and sex factors. The MCI vs. HC comparison maintained stable age/sex distributions, precluding additional stratification. In AD vs. HC CSF comparisons, the subgroup with <50% female participants showed non-significant differences, potentially reflecting inadequate power. Notably, blood sample stratification revealed distinct patterns: serum YKL-40 showed no group-wise differences in AD vs. HC or MCI vs. HC comparisons, whereas plasma YKL-40 effectively discriminated both AD from HC and MCI from HC. This aligns with previous findings that serum YKL-40 correlates with CSF levels and structural MRI parameters but not with cognitive performance or clinical staging (55). It should be emphasized that the limited sample size of blood YKL-40 studies in our analysis may introduce measurement variability.

### 4.2 YKL-40 and astrocyte-driven neuroinflammation in AD

Increasing evidence identifies neuroinflammation as a key hallmark of AD pathophysiology. Microglia and astrocytes, acting as the principal cellular mediators of these inflammatory processes, are instrumental in the progression of AD throughout both the preclinical and symptomatic stages (56). Studies on human brain pathology confirm that YKL-40 is synthesized by a subset of astrocytes in AD patients (57). Its colocalization with tau immunoreactivity suggests that YKL-40 levels directly reflect the intensity of the AD pathological cascade, providing a biological basis for its use as a progression biomarker.

YKL-40 is a protein predominantly expressed in astrocytes and encoded by the CHI3L1 gene. CHI3L1 is primarily expressed by astrocytes in the mouse brain, with elevated expression detected in reactive astrocytes in advanced AD, accompanied by increased IBA1-positive microglial activation (58). Emerging evidence demonstrates that astrocyte-specific YKL-40 knockout in symptomatic 5xFAD mice with cognitive deficits effectively decreases amyloid plaque deposition in multiple brain regions, along with reduced glial activation, less neuronal loss, and improved memory function (59–61). These findings are further supported by *in vitro* evidence, which reveals that pro-inflammatory cytokines such as IL-1, IL-6, and TNF-*α*, alongside M1-type macrophage secretomes, upregulate CHI3L1 expression in astrocytes (62). These molecular findings confirm the pivotal role of reactive astrocytes in the neuroinflammatory cascade associated with AD, positioning YKL-40 as a key biomarker for reactive astrocytes. They further reinforce the potential application value of YKL-40 as a biomarker for monitoring disease progression in AD.

### 4.3 Clinical implications and translational potential of blood-based YKL-40

CSF can effectively reflect pathological changes in brain tissue due to its direct contact. Although CSF is considered an optimal measure for AD, its acquisition requires invasive lumbar puncture, which underlies the urgent need for less invasive diagnostic techniques (63). In recent years, increasing attention has been directed toward blood-based biomarkers as accessible and scalable tools for assessing AD–related pathology. Compared with CSF sampling, plasma biomarkers offer clear practical advantages, including lower invasiveness, improved feasibility for large-scale screening, and suitability for longitudinal monitoring (64). Notably, an umbrella reanalysis of 106 meta-analyses encompassing 52 candidate biomarkers identified blood YKL-40 as the biomarker achieving Class I (convincing) evidence for association with AD (65). Existing evidence confirms that plasma YKL-40 correlates with CSF YKL-40 levels, with correlation coefficients ranging from low (*r* = 0.24) to moderate (*r* = 0.40) (66, 67). These findings support the potential of plasma YKL-40 as a peripheral indicator of neuroinflammatory activity related to AD progression, while highlighting its value as a more accessible biomarker for population-based and longitudinal studies.

Specifically, our subgroup analysis demonstrated that plasma YKL-40, rather than serum, serves as a more robust indicator for differentiating AD and MCI from healthy controls. This finding not only aligns with the clinical shift toward non-invasive diagnostics but also provides practical evidence for optimizing sample selection in future clinical applications. Admittedly, individual heterogeneity caused by factors such as age, sex, and comorbidities can affect disease development, and plasma YKL-40 is susceptible to these confounding factors, which may compromise diagnostic specificity when used alone. Therefore, plasma YKL-40 should be considered an adjunctive biomarker rather than a standalone diagnostic tool, and its utility may be enhanced when integrated into multimodal frameworks (e.g., combined with plasma p-tau or MRI parameters) to mitigate the impact of systemic confounders.

### 4.4 Association of YKL-40 with tau pathology in AD

Accumulating evidence indicates that CSF YKL-40 levels are positively associated with tau pathology, while showing no significant correlation with amyloid-*β* (Aβ) burden, demonstrating strong associations with tau pathophysiology even during preclinical AD stages (68). Previous studies have reported that YKL-40 is released into the CSF at a relatively later stage of the AD pathological cascade and may specifically mediate the link between Aβ-induced downstream tau phosphorylation and tau-related neurodegeneration (47). Neuroimaging-pathology correlation studies further reveal spatially specific positive relationships between CSF YKL-40 levels and tau accumulation in AD-vulnerable regions (e.g., hippocampus), independent of Aβ deposition (68). Recent studies have further demonstrated consistent findings showing significant correlations between CSF YKL-40 and both phosphorylated tau (p-tau) and total tau (t-tau), with particularly strong associations observed in A + MCI and T + subgroups (69). Evidence shows that YKL-40 may be involved in the non-amyloid-dependent pathway. Elevated levels of YKL-40 in the cerebrospinal fluid were observed in A + T + and A-T + individuals, whereas such elevation was not detected in A + T- individuals (47, 68, 70, 71). Intriguingly, a structural equation model demonstrates that Aβ fibril aggregates directly trigger astrocytic (47). Quantitative Aβ-PET studies reveal that higher CSF YKL-40 levels correlate with greater global Aβ burden and reduced hippocampal volume, findings replicated across another cohort (52, 72). Crucially, mediation analysis demonstrates these phenotypic associations are fully mediated by p-tau levels.

### 4.5 Limitations

This study has several methodological limitations: First, although the overall sample size was substantial, the number of studies and participants in certain subgroups, particularly those involving preclinical AD and peripheral blood measurements, remains limited. Thereby constraining the evaluation of YKL-40’s discriminative capacity during the ultra-early transitional stages of AD. Second, key confounders, including comorbid inflammatory conditions, APOE genotypes, and Aβ pathological status, were inadequately documented in most original studies, potentially introducing residual confounding bias. Notably, significant geographic constraints exist—most of the participants originated from Western populations—a sampling bias that may jeopardize the generalizability of conclusions. These limitations underscore the need for future large-scale, longitudinal studies with standardized biomarker measurements to further validate the role of YKL-40 across the Alzheimer’s disease continuum.

## 5 Conclusion

This study revealed two key findings: (1) CSF YKL-40 levels demonstrate progressive elevation across AD, MCI, and pre-AD groups; (2) Peripheral blood YKL-40, particularly plasma measurements, shows significant increases in AD and MCI patients compared to HCs. These results establish CSF YKL-40 as a diagnostically relevant biomarker for early AD detection and progression monitoring, while plasma YKL-40 emerges as a non-invasive discriminator of AD/MCI from normal aging. However, the observed limitations—moderate diagnostic specificity and susceptibility to systemic inflammatory confounders—highlight the necessity of developing multi-analyte panels incorporating complementary inflammatory markers to enhance staging accuracy and progression tracking. Despite these constraints, our findings provide critical evidence for YKL-40’s biomarker potential through its dynamic changes along the AD pathological spectrum. Crucially, large-scale multicenter studies are imperative to validate the synergistic diagnostic value of glial activation markers like sTREM2 and GFAP with YKL-40, thereby advancing molecular surveillance frameworks for AD progression.

## Data Availability

The original contributions presented in the study are included in the article/Supplementary material, further inquiries can be directed to the corresponding author.
