# Supplementary material for: Association of Alzheimer’s disease progression with YKL40 levels in peripheral blood and cerebrospinal fluid: a systematic review and meta-analysis
Source: Front Neurol. 2026 Feb 5;17:1768353. doi: 10.3389/fneur.2026.1768353 (PMC12916370; doi:10.3389/fneur.2026.1768353)
Supplement: Supplementary file 1 [file Table_1.docx]

Supplementary Material

# Supplementary Tables

**Supplementary Table S1. Search strategy**

| Datebase | Number | Search terms | Items |
| --- | --- | --- | --- |
| Pubmed | #1 | YKL-40[Title/Abstract] OR CHI3L1[Title/Abstract] OR cartilage gp39[Title/Abstract] OR GP39[Title/Abstract] OR glycoprotein-39[Title/Abstract] OR 38-kDa heparin-binding glycoprotein[Title/Abstract] OR chitinase 3-like 1[Title/Abstract] OR HC-gp39 protein[Title/Abstract] | 2614 |
|  | #2 | Alzheimer’s disease[Title/Abstract] OR Alzheimer[Title/Abstract] OR Alzheimer-Type Dementia[Title/Abstract] OR Senile Dementia[Title/Abstract] OR Alzheimer*[Title/Abstract] OR Primary Senile Degenerative Dementia[Title/Abstract] OR Alzheimer Sclerosis[Title/Abstract] OR Cognition disorders[Title/Abstract] OR AD[Title/Abstract] OR Mild Cognitive Impairment[Title/Abstract] OR MCI[Title/Abstract] | 336,494 |
|  | #3 | #1 AND #2 | 256 |
| Medline | #1 | XB YKL-40 OR XB CHI3L1 OR cartilage gp39 OR OR GP39 OR glycoprotein-39 OR 38-kDa heparin-binding glycoprotein OR chitinase 3-like 1 OR HC-gp39 protein | 2299 |
|  | #2 | XB Alzheimer’s disease OR Alzheimer OR Alzheimer-Type Dementia OR Senile Dementia OR Alzheimer* OR Primary Senile Degenerative Dementia OR Alzheimer Sclerosis OR Cognition disorders OR AD OR Mild Cognitive Impairment OR MCI | 319440 |
|  | #3 | #1 AND #2 | 242 |
| Cochrane library | #1 | (YKL-40):ti,ab,kw OR (CHI3L1):ti,ab,kw OR (cartilage gp39):ti,ab,kw OR (GP39):ti,ab,kw OR (glycoprotein-39):ti,ab,kw OR (HC-gp39 protein):ti,ab,kw | 245 |
|  | #2 | (Alzheimer’s disease):ti,ab,kw OR (Alzheimer):ti,ab,kw OR (Alzheimer-Type Dementia):ti,ab,kw OR (Senile Dementia):ti,ab,kw OR (Alzheimer*):ti,ab,kw OR (Primary Senile Degenerative Dementia):ti,ab,kw OR (Alzheimer Sclerosis):ti,ab,kw OR (Cognition disorders):ti,ab,kw OR (Mild Cognitive Impairment):ti,ab,kw OR (AD):ti,ab,kw OR (MCI):ti,ab,kw | 46504 |
|  | #3 | #1 AND #2 | 43 |
| Web of science | #1 | TS=(YKL-40) OR TS=(CHI3L1) OR TS=(cartilage gp39) OR TS=(GP39) OR TS=(glycoprotein-39) OR TS=(38-kDa heparin-binding glycoprotein) OR TS=(chitinase 3-like 1) OR TS=(HC-gp39 protein) | 4549 |
|  | #2 | TS=(Alzheimer’s disease) OR TS=(Alzheimer) OR TS=(Senile Dementia) OR TS=(Alzheimer-Type Dementia) OR TS=(Alzheimer*) OR TS=(Primary Senile Degenerative Dementia) OR TS=(Alzheimer Sclerosis) OR TS=(Cognition disorders) OR TS=(AD) OR TS=(Mild Cognitive Impairment) OR TS=(MCI) | 1121833 |
|  | #3 | #1 AND #2 | 444 |

Supplementary Table S2. Characteristics of the included studies (CSF) for the meta-analysis

| Study | Year | Country | Diagnostic criteria | Mean age (years) | Population | n | Women/men | YKL40 levels (ng/ml) | Assay method | Sample type | Quality Score |
| --- | --- | --- | --- | --- | --- | --- | --- | --- | --- | --- | --- |
| Agnieszka Kulczy-nska-Przybik | 2023 | Poland | NIA-AA | NA | AD | 40 | 31:9 | 396.87 (190.69) | ELISA | CSF | 6 |
|  |  |  |  | NA | MCI | 18 | 10:8 | 415.11 (159.28) |  |  |  |
|  |  |  |  | NA | HC | 20 | 12:8 | 298.48 (108.51) |  |  |  |
| Linbin Dai | 2023 | China | NIA-AA | 63.8 (7.25) | AD | 78 | 28:50 | 282 (107) | ELISA | CSF | 8 |
|  |  |  |  | 66.0 (6.56) | MCI | 37 | 16:21 | 298 (115) |  |  |  |
|  |  |  |  | 60.3 (8.35) | HC | 58 | 29:29 | 196 (64) |  |  |  |
| Anna M De Kort | 2021 | Netherlands | NINCDS-ADRDA | 67 (7) | AD | 17 | 9:8 | 323 (40) | ELISA | CSF | 7 |
|  |  |  |  | 70 (3) | MCI | 33 | 23:10 | 324 (63) |  |  |  |
|  |  |  |  | 63 (4) | HC | 50 | 25:25 | 226 (51) |  |  |  |
| Agnieszka Kulczyńska-Przybik | 2020 | Poland | NIA-AA | NA | AD | 42 | NA | 401.15 (97.9) | ELISA | CSF | 6 |
|  |  |  |  | NA | MCI | 18 | NA | 438.57 (144.09) |  |  |  |
|  |  |  |  | NA | HC | 20 | NA | 289.31 (65.03) |  |  |  |
| Masaki Ikeda | 2020 | Japan | NIA-AA | 69 (1.49) | AD | 24 | 13:11 | 107.23 (10.26) | ELISA | CSF | 7 |
|  |  |  |  | 65 (2.34) | HC | 18 | 9:9 | 60.53 (5.43) |  |  |  |
| Anna Antonell | 2019 | Spain | NIA-AA | 63.21 (8.04) | AD | 108 | 68:40 | 316.96 (112.13) | ELISA | CSF | 7 |
|  |  |  |  | 68.20 (8.98) | MCI | 56 | 36:20 | 333.20 (106.03) |  |  |  |
|  |  |  |  | 69.15 (9.78) | pre-AD | 21 | 14:7 | 307.42 (100.56) |  |  |  |
|  |  |  |  | 56.82 (13.21) | HC | 50 | 34:16 | 213.96 (89.70) |  |  |  |
| Nicola Toschi | 2019 | USA | NINCDS-ADRDA | 70.4 (7.7) | AD | 37 | 19:18 | 153.36 (54.31) | ELISA | CSF | 7 |
|  |  |  |  | 71.7 (8.4) | MCI | 20 | 7:13 | 145.37 (57.04) |  |  |  |
|  |  |  |  | 60.7 (10.3) | HC | 20 | 13:7 | 102.91 (40.90) |  |  |  |
| Alberto Lleó | 2019 | Spain | NIA-AA | 68.5 (8.5) | AD | 110 | 47:63 | 209.73 (72.76) | ELISA | CSF | 7 |
|  |  |  |  | 67 (8.4) | MCI | 128 | 49:79 | 175.99 (74.55) |  |  |  |
|  |  |  |  | 58.2 (7.2) | HC | 154 | 86:68 | 131.61 (49,54) |  |  |  |
| Kaja Nordengen | 2019 | Norway | NIA-AA | 67.6 (5.2) | AD | 27 | 13:14 | 221 (70) | ELISA | CSF | 7 |
|  |  |  |  | 66.6 (7.4) | MCI | 40 | 23:17 | 182 (69) |  |  |  |
|  |  |  |  | 61.1 (9.2) | HC | 36 | 19:17 | 145 (46) |  |  |  |
| Hua Zhang | 2018 | China | NINCDS-ADRDA | 74.3 (1.6) | AD | 18 | 11:7 | 471.9 (39.5) | ELISA | CSF | 8 |
|  |  |  |  | 76.7 (1.1) | MCI | 24 | 7:17 | 384.0 (28.1) |  |  |  |
|  |  |  |  | 76.0 (1.0) | HC | 32 | 13:19 | 397.2 (25.7) |  |  |  |
| Harald Hampel | 2018 | France | NINCDS-ADRDA | 72.29 (6.18) | AD | 35 | 24:11 | 147.42 (44.83) | ELISA | CSF | 7 |
|  |  |  |  | 70.58 (7.68) | MCI | 41 | 14:27 | 137.22 (66.07) |  |  |  |
|  |  |  |  | 64 (7.95) | HC | 21 | 13:8 | 99.44 (15.90) |  |  |  |
| Franc Llorens | 2017 | Germany | NINCDS-ADRDA | 67 (11) | AD | 65 | 43:22 | 400 (181) | ELISA | CSF | 8 |
|  |  |  |  | 70 (6) | HC | 50 | 27:23 | 254 (117) |  |  |  |
| Shorena Janelidze | 2015 | Sweden | NINCDS-ADRDA | 75.0 (7.6) | AD | 35 | 23:12 | 219 (59) | ELISA | CSF | 8 |
|  |  |  |  | 69.2 (7.5) | MCI | 62 | 35:27 | 184 (69) |  |  |  |
|  |  |  |  | 75.3 (6.4) | HC | 53 | 37:16 | 200 (64) |  |  |  |
| Maartje I Kester | 2015 | Netherlands | NINCDS-ADRDA | 65 (1) | AD | 65 | 29:36 | 288 (12) | ELISA | CSF | 7 |
|  |  |  |  | 68 (1) | MCI | 61 | 23:38 | 304 (16) |  |  |  |
|  |  |  |  | 64 (2) | HC | 37 | 14:23 | 231 (16) |  |  |  |
| Daniel Alcolea | 2014 | Spain | NINCDS-ADRDA | 70.65 (9.88) | AD | 59 | 38:21 | 260.35 (55.47) | ELISA | CSF | 7 |
|  |  |  |  | 70.71 (7.66) | MCI | 45 | 22:23 | 258.80 (50.54) |  |  |  |
|  |  |  |  | 63.28 (9.46) | HC | 24 | 13:11 | 206.73 (59.89) |  |  |  |
| Bob Olsson | 2013 | Sweden | NINCDS-ADRDA | 76.2 (7.4) | AD | 96 | 62:34 | 241.58 (100.70) | ELISA | CSF | 7 |
|  |  |  |  | 68.8 (7.3) | MCI | 81 | 44:37 | 171.69 (68.37) |  |  |  |
|  |  |  |  | 74.7 (7.5) | HC | 65 | 48:17 | 194.62 (76.33) |  |  |  |
| Niklas Mattsson | 2011 | Sweden | NINCDS-ADRDA | 74 (4) | AD | 25 | 14:11 | 223 (70) | ELISA | CSF | 8 |
|  |  |  |  | 73 (4) | pre-AD | 7 | 3:4 | 193 (30) |  |  |  |
|  |  |  |  | 74 (5) | HC | 19 | 10:9 | 198 (52) |  |  |  |
| Samir Abu-Rumeileh | 2019 | Italy | IWG-2 | 68.63 (8.16) | AD | 40 | 16:24 | 236.10 (89.96) | ELISA | CSF | 8 |
|  |  |  |  | 64.88 (9.62) | HC | 40 | 18:22 | 140.03 (35.37) |  |  |  |
| Estrella Morenas-Rodríguez | 2019 | Spain | NIA-AA | 74.6 (5.6) | AD | 50 | 31:19 | 295.3 (54.1) | ELISA | CSF | 7 |
|  |  |  |  | 72.3 (6.3) | pre-AD | 53 | 32:21 | 296.7 (55.7) |  |  |  |
|  |  |  |  | 67.4 (5.1) | HC | 44 | 25:19 | 238.8 (49.2) |  |  |  |
| Estrella Morenas-Rodr´ıguez | 2016 | Spain | NIA-AA | 70 (8.4) | AD | 73 | 45:28 | 267.5 (52.8) | ELISA | CSF | 7 |
|  |  |  |  | 67.9 (8.7) | MCI | 90 | 49:41 | 251.1 (49.8) |  |  |  |
|  |  |  |  | 59 (8.3) | HC | 74 | 51:23 | 195.1 (44.6) |  |  |  |
| Anna Antonell | 2014 | Spain | NIA-AA | 71.8 (8.6) | pre-AD | 18 | 9:9 | 330.0 (120.1) | ELISA | CSF | 7 |
|  |  |  |  | 61.6 (7.6) | HC | 43 | 31:12 | 260.5 (71.6) |  |  |  |
| Christina Manniche | 2020 | Denmark | NIA-AA | 70.28 (8.0) | AD | 57 | 27:30 | 182 (70) | ELISA | CSF | 7 |
|  |  |  |  | 64.52 (7.6) | HC | 33 | 14:19 | 150 (48) |  |  |  |
| Daniel Alcolea | 2017 | Spain | NIA-AA | 70.8 (7.8) | AD | 72 | 44:28 | 280.3 (47.6) | ELISA | CSF | 7 |
|  |  |  |  | 60.2 (8.3) | HC | 76 | 45:31 | 199.8 (50.3) |  |  |  |
| Isabel Schulz | 2021 | Germany | NIA-AA | 74.27 (4.64) | AD | 11 | 5:6 | 225.76 (121.34) | ELISA | CSF | 7 |
|  |  |  |  | 68.75 (6.38) | HC | 20 | 6:14 | 146.55 (42.41) |  |  |  |

Supplementary Table S3. Characteristics of the included studies (peripheral blood) for the meta-analysis

| Study | Year | Country | Diagnostic criteria | Mean age (years) | Population | n | Women/men | YKL40 levels (ng/ml) | Assay method | Sample type | Quality Score |
| --- | --- | --- | --- | --- | --- | --- | --- | --- | --- | --- | --- |
| Anja Soldan | 2025 | Moldova | NIA-AA | 54.9 (9.5) | MCI | 82 | 50:32 | 60.4 (56.0) | Elecsys | Plasma | 7 |
|  |  |  |  | 54.9 (9.5) | HC | 189 | 114:75 | 42.0 (31.6) |  |  |  |
|  |  |  |  | 69.1 (5.9) | MCI (2) | 31 | 21:10 | 97.4 (115.5) |  |  |  |
|  |  |  |  | 63.7 (9.1) | HC (2) | 171 | 105:66 | 63.9 (66.7) |  |  |  |
| Inès Schmidt-Morgenroth | 2023 | Switzerland | NIA-AA | 69.1 (8.3) | MCI | 32 | 20:12 | 66.73 (60.43) | simple-plex | Serum | 7 |
|  |  |  |  | 59.6 (6.1) | HC | 30 | 15:15 | 67.05 (69.11) |  |  |  |
| Fumihiko Yasuno | 2022 | Japan | NIA-AA | 78.1 (3.9) | AD | 15 | 8:7 | 97.8 (62.8) | ELISA | Serum | 8 |
|  |  |  |  | 78.9 (5.2) | HC | 10 | 6:4 | 89.7 (66.2) |  |  |  |
| Pan-Woo Ko | 2021 | Korea | NIA-AA | 65.2 (9.9) | AD | 121 | 70:51 | 87.15 (68.01) | ELISA | Plasma | 7 |
|  |  |  |  | 68.2 (10.0) | MCI | 23 | 12:11 | 62.16 (61.2) |  |  |  |
|  |  |  |  | 64.7 (9.9) | HC | 83 | 47:36 | 56.65 (34.68) |  |  |  |
| Agnieszka Kulczyńska-Przybik | 2020 | Poland | NIA-AA | NA | AD | 42 | NA | 66.98 (30.57) | ELISA | Blood | 7 |
|  |  |  |  | NA | MCI | 18 | NA | 73.69 (39.80) |  |  |  |
|  |  |  |  | NA | HC | 20 | NA | 69.12 (25.43) |  |  |  |
| Anna Villar-Piqué | 2019 | Germany | NIA-AA | 69 (10) | AD | 50 | 25:25 | 133 (110) | ELISA | Plasma | 8 |
|  |  |  |  | 66 (5) | HC | 70 |  | 84 (84) |  |  |  |
| Jihye Choi | 2011 | Korea | NINCDS-ADRDA | 68 (1.00) | MCI | 49 | 30:19 | 176.49 (25.68) | ELISA | Plasma | 7 |
|  |  |  |  | 63.88 (0.96) | HC | 35 | 22:13 | 96.91 (11.02) |  |  |  |
| Isabel Schulz | 2021 | Germany | NIA-AA | 74.27 (4.64) | AD | 11 | 5:6 | 92.85 (55.06) | ELISA | Serum | 7 |
|  |  |  |  | 68.75 (6.38) | HC | 20 | 6:14 | 66.05 (55.36) |  |  |  |
